# Comparison of Gefitinib in the treatment of patients with non-small cell lung cancer and clinical effects of Osimertinib and EGFR Gene mutation

**DOI:** 10.12669/pjms.38.6.5456

**Published:** 2022

**Authors:** Xiaofeng Li, Zhanqiang Zhai, Youcai Zhu, Haiou Zhou

**Affiliations:** 1 Xiaofeng Li Department of Thoracic Disease Center, Zhejiang Rongjun Hospital, 309 Shuangyuan Road, Jiaxing 314000, Zhejiang Province, P.R. China; 2 Zhanqiang Zhai, Department of Thoracic Disease Center, Zhejiang Rongjun Hospital, 309 Shuangyuan Road, Jiaxing 314000, Zhejiang Province, P.R. China; 3 Youcai Zhu, Department of Thoracic Disease Center, Zhejiang Rongjun Hospital, 309 Shuangyuan Road, Jiaxing 314000, Zhejiang Province, P.R. China; 4 Haiou Zhou, Department of Anesthesiology, Zhejiang Rongjun Hospital, 309 Shuangyuan Road, Jiaxing 314000, Zhejiang Province, P.R. China

**Keywords:** Non-small cell lung cancer, Epidermal growth factor receptor, Targeted therapy, Osimertinib, Gefitinib

## Abstract

**Objectives:**

To compare the clinical effects of Osimertinib and Gefitinib in the treatment of non-small cell lung (NSCLC) complicated with epidermal growth factor receptor (EGFR) gene mutation.

**Methods:**

We retrospectively analyzed the clinical data of 102 patients with advanced NSCLC and EGFR gene mutations treated in the Chest Disease Diagnosis and Treatment Center of our hospital from January 2018 to January 2020. We divided the data based on the administered treatments into Osimertinib and Gefitinib groups. The disease control rate (DCR), progression free survival (PFS) and the incidence of adverse events in both groups were analyzed.

**Results:**

In the Osimertinib group, there was one patients with complete response (CR), 38 with partial response (PR), eight with stable disease (SD), and two with progressive disease (PD)/ The overall response rate (ORR) was 79.59% (39/49), and the disease control rate (DCR) was 95.92% (47/49). In the Gefitinib group, we found zero patients with CR, 37 patients with PR, 11 with SD, and five with PD. The ORR in the Gefitinib group was 69.80% (37/53) and DCR was 90.57% (48/53). There was no statistical significance between the two groups, ORR was Χ^2^=0.927 (P=0.336) and the DCR Χ^2^=0.221 (P=0.638). The median PFS of and Gefitinib groups was significantly higher in the oxitinib group, compared to the Gefitinib group (18.1 months (95% CI 15.4-20.7) and 10.7 months (95% CI 9.9-11.4), respectively, P<0.001). The incidence of adverse reactions in the Osimertinib group was 12.24% (6/49), which was significantly lower than 28.30% (15/53) in the Gefitinib group (P < 0.05).

**Conclusions:**

The clinical effect of oxitinib in the treatment of non-small cell lung cancer complicated with EGFR gene mutation is similar to that of Gefitinib. In patients with advanced NSCLC and EGFR gene mutations, oxitinib treatment is associated with significantly longer PFS and lower adverse reaction rate compared with Gefitinib treatment.

## INTRODUCTION

Lung cancer is one of the most common and frequently diagnosed malignancies and is considered a leading cause of cancer-related mortality worldwide, with about two million new cases reported annually. Lung cancer mortality reaches as high as 18.4%, significantly higher rate than that of the second ranked breast cancer (6.6%).^1^ In China, the incidence rate of lung cancer is 20%, with a mortality rate of 27.3%.^2^ About 80% of lung cancers patients are diagnosed with non-small cell lung cancers (NSCLCs) with a poor prognosis. More than half of patients with NSCLC are diagnosed at the advanced stage which makes surgery impossible.^3^ Numerous studies showed the association of mutations in epidermal growth factor receptor (EGFR) with NSCLC.^4^ Therefore, EGFR tyrosine kinase inhibitors (TKIs) have been recommend as a potential first-line treatment for advanced NSCLC patients with EGFR mutations positive.^5^ However, acquisition of an additional mutation in EGFR, resulting in substitution of threonine amino acid 790 to methionine (T790M) is the most common resistance mechanism that occurs.^6^,^7^

Osimertinib (trade name Tagrisso) is a third-generation EGFR-TKI developed by AstraZeneca in the UK. It can target both the initial EGFR mutation and the T790M site mutation at the same time, and it is used to treat patients with NSCLC and the EGFR T790M mutation after receiving EGFR-TKI treatment.^8^,^9^ In March 2017, the U.S. Food and Drug Administration (FDA) approved Osimertinib as a first-line treatment for patients with metastatic NSCLC with EGFR mutation (deletion of exon 19 or mutation of exon 21 L858R), and obtained the permission of first-line treatment in China in August 2019.^10^,^11^

Gefitinib (trade name Iressa) is a reversible small molecule ATP analogue that is designed to inhibit the tyrosine kinase activity of EGFR. It is a first molecular target medication developed by AstraZeneca for the treatment of NSCLC.^12^ Compared with traditional chemotherapy drugs, Gefitinib, a targeted drug that regulates tumor pathogenesis at the molecular level of cell receptor and value-added regulation, can significantly prolong the disease-free survival of patients with EGFR mutation positive.^13^

This study investigated and compared clinical effect and adverse reaction rate of Oxitinib and Gefitinib in the treatment of non-small cell lung cancer complicated with EGFR gene mutation to provide reference for relevant clinical application.

## METHODS

We retrospectively collected data from 102 patients with stage III-B or IV NSCLC treated at the Chest Disease Diagnosis and Treatment Center of our hospital from January 2018 to January 2020. According to clinical records, 49 patients were treated with Osimertinib and 53 with Gefitinib.

### Inclusion criteria:

• EGFR mutation was determined by molecular pathology, and the mutation status of exons 19 and 21 of EGFR mutation was detected by ARMS-PCR;^14^
• There is at least one objectively measurable tumor focus, and the maximum diameter measured by conventional measurement technology is ≥20 mm;
• The clinical data are complete.

### Exclusion criteria:

• Serious functional organ damage;
• Patients with mental disorders;
• Severe cardiac insufficiency.

Patients were treated with either Oxitinib (AstraZeneca Pharmaceutical Co., Ltd., gyzz j20180027), 80mg/tablet orally, one tablet/day, or Gefitinib (AstraZeneca Pharmaceutical Co., Ltd., gyzz j20180014), 250mg/tablet orally, one tablet/day.

Each treatment was given as a cycle of one month. The drug was administered continuously during the treatment until the disease progresses or intolerable adverse reactions occur. Patients did not receive radiotherapy or other molecular targeted drugs during the whole treatment period.

### Observation indexes and curative effect evaluation criteria

1) General clinical data of patients were collected; 2) Disease control: the clinical effects of patients before and after treatment were evaluated according to RECIST solid tumor efficacy evaluation standard.^15^ Designed to better capture CIT responses. Patients and Methods Atezolizumab data from clinical trials in nonsmall- cell lung cancer, metastatic urothelial carcinoma, renal cell carcinoma, and melanoma were evaluated. Modifications to imRECIST versus RECIST v1.1 included allowance for best overall response after progressive disease (PD At the end of the treatment cycle, computer tomography (CT) was performed using Siemens SOMATOM spirit multi-slice spiral CT to examine the area from the apex to the bottom of the lung. The scanning parameters were as follows: pitch 1.08, voltage 120 kV, current 250Ma, reconstruction layer thickness 5mm, scanning time 5~7s, matrix 512×512. At the same time, high-resolution target scanning was carried out. The scanning parameters were: pitch 0.64, voltage 120kV, current 300 Ma, reconstruction layer thickness 2mm, scanning time 5~7s, matrix 1024×1024, calculated according to the high-resolution algorithm.^16^ The evaluation criteria are shown in Table-I; 3)Occurrence of adverse reactions, mainly including bone marrow suppression, rash, gastrointestinal reaction, pulmonary interstitial fibrosis and other adverse reactions.

**Table I:** Evaluation criteria for the efficacy of the treatment on solid tumors.

Type	Concrete content
Complete remission (CR)	The target lesions disappeared completely, and no new lesions were apparent for ≥4 weeks
Partial remission (PR)	The total reduction of the target lesion diameter was ≥30%, and no new lesions appeared for ≥4 weeks
Stability disease (^SD)^	The total diameter of the target lesions decreased <30% or increased <20%
Progressive disease (PD)	In addition to the target lesions, metastasis, new lesions, or the total diameter of the target lesions increased more than 20%
Objective response rate (ORR)	CR+PR
Disease control rate (DCR)	CR+PR+SD

Follow-ups were conducted in the outpatient clinic or by telephone. PFS was defined as the time from the beginning of the patient’s medication treatment to the time defined as PD.

### Statistical Analysis

We used the Spss22.0 software for statistical analyses. We used the χ^2^ test to analyze general data and clinical efficacy between groups and logistic regression to analyze the influencing factors of ORR. In addition, we applied the Kaplan Meier method and Cox model to analyze PFSs and OSs (significance level α=0.05).

### Ethical approval

The medical ethics committee of our hospital approved the study plan (approval number 2018-056; Nov 15, 2019).

## RESULTS

**Fig.1:**
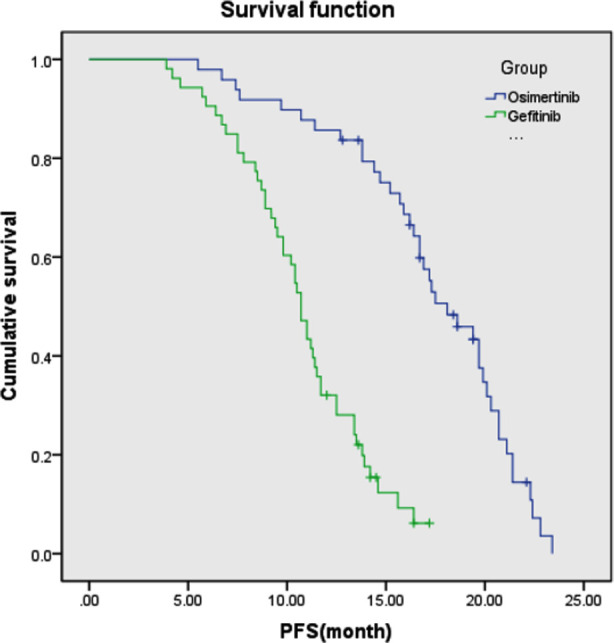
Progression free survival (PFS) curves in the two groups of patients with non-small celllung cancer.

We found no significant difference in gender, age, smoking status, PS score, CNS metastasis, pathological type and clinical stage between the two groups (Table-II). In the Osimertinib group, we found one patient with CR, 38 with PR, eight with SD, and two with PD; The ORR 79.59% (39/49) and DCR was 95.92% (47/49). In the Gefitinib group, we found zero patients with CR, 37 with PR, 11 with SD, and five with PD; the ORR 69.80% (37/53) and DCR was 90.57% (48/53), there was no statistical significance between the two groups, ORR was χ^2^=0.927 (P=0.336) and the DCR χ^2^=0.221 (P=0.638), but it was higher in the Osimertinib group than those in the Gefitinib group (Table-III). The median PFS of oxitinib and Gefitinib groups were 18.1 months (95% CI 15.4-20.7) and 10.7 months (95% CI 9.9-11.4), respectively, the difference was statistically significant (P < 0.001) (Fig.1). The incidence of adverse reactions in the Osimertinib group was 12.24% (6 / 49), which was significantly lower than 28.30% (15/53) of Gefitinib (P < 0.05) (Table-IV).

**Table II:** Comparison of general data between the two groups (49/53).

Group	Sex (M/F, n)	Age (≤65/>65, n)	Smoke status (Y/N, n)	PS score (<2/≥2, n)	CNS transfer (Y/N, n)	Pathological type				TNM	

						Squamous cell carcinoma	Adenocarcinoma	Large cell carcinoma	Others	IIIb	IV
Osimertinib	24/25	27/22	38/11	34/15	41/8	1	48	0	0	30	19
Gefitinib	26/27	25/28	35/18	40/13	37/16	0	53	0	0	35	18
χ²	0	0.641	1.659	0.214	2.719	1.092				0.255	
P	0.994	0.423	0.198	0.664	0.099	0.296				0.613	

**Table III:** Comparison of clinical efficacy between the two groups [n (%)].

Group	CR	PR	SD	PD	ORR	DCR
Osimertinib group(n=49)	1(2.04)	38(77.55)	8(16.33)	2(4.08)	39(79.59)	47(95.92)
Gefitinib group(n=53)	0(0)	37(69.81)	11(20.75)	5(9.43)	37(69.81)	48(90.57)
Χ^2^					0.221	0.927
P					0.638	0.336

**Table IV:** Comparison of adverse reaction rates between the two groups [n (%)].

Group	Myelosuppression	Rash	Digestive tract reaction	Pulmonary interstitial fibrosis	Incidence of adverse reactions
Osimertinib group(n=49)	1(2.04)	2((4.08))	2((4.08))	1(4.08)	6(12.24)
Gefitinib group(n=53)	3(5.66)	4(7.55)	5(9.43)	3(5.66)	15(28.30)
Χ^2^					4.015
P					0.045

## DISCUSSION

EGFR mutations have been detected in about 14% of patients with non-small cell lung cancer, EGFR-TKI can inhibit signal transduction by blocking the phosphorylation process of tyrosine kinase region, so as to play an anti-tumor role.^17^,^18^ In recent years, erlotinib and Gefitinib are molecular targeted drugs with high application frequency, as a kind of EGFR-TKI, they can produce certain antitumor effects by preventing EGFR phosphorylation and inhibiting signal transduction such as proliferation and apoptosis of tumor cells. However, relevant studies have shown that the third generation EGFR-TKI targeted drugs represented by Osimertinib can prolong the survival time of 50%~60% of patients with T790M mutation and drug resistance.^19^ Yamada Y et al. showed that the PFS of patients with EGFR gene mutation in non-small cell lung cancer treated with Osimertinib was significantly longer than that of Gefitinib.^20^ Park S et al. also showed that osimitinib showed good Orr and survival benefits and high safety in the treatment of non-small cell lung cancer complicated with EGFR gene mutation.^21^ The results of this study show that the ORR and DCR of Osimertinib group are equivalent to that of Gefitinib group, but the PFS is significantly longer than that of Gefitinib group, and the adverse reaction rate is lower, which is consistent with the relevant research results.

With the continuous research of molecular targeted drug therapy, especially the continuous renewal and development of the first and second generation EGFR tyrosine kinase inhibitors, the progression free survival of patients with non-small cell lung cancer complicated with EGFR gene mutation has been greatly improved, but most patients have developed drug resistance within 1~2 years of treatment, which inevitably leads to the progression of tumor.^22^ In recent years, relevant studies have found that the permeability of blood-brain barrier is considered to be an ideal choice for targeted drugs to improve the clinical efficacy of non-small cell lung cancer. However, due to the different molecular configuration and permeability of these drugs to blood-brain barrier, their activity in the treatment of patients with brain metastasis of non-small cell lung cancer is limited.^23^ Oxitinib is the third generation of EGFR tyrosine kinase inhibitor, which can selectively inhibit L858R / T790M EGFR mutation. Compared with other EGFR-TKIs, its efficacy in the brain is significantly enhanced. It is the third generation of efficient and selective irreversible oral EGFR-TKI following Gefitinib and afatinib, which can effectively and selectively inhibit T790M mutation and EGFR sensitive mutation, at the same time, it retains the activity of wild-type EGFR in normal tissues, so as to reduce its own toxicity.^24^,^25^ In the meta-analysis of Wang N et al, eleven studies were included, including 842 patients, including four randomized controlled trials, three single arm studies, two retrospective studies and two real-world studies. It was found that the Orr and DCR of patients with brain metastatic lung cancer with EGFR mutation were 71% and 93% respectively.^26^ In addition, Japanese scholar Ohe Y et al. In a multicenter study, the median progression free survival of osimetinib and Gefitinib were 19.1 and 13.8 months, respectively.^27^

This study shows that in terms of adverse reactions of drug treatment, the incidence of adverse reactions in the Osimertinib group after treatment is significantly lower than that in the Gefitinib group. The mechanism of action of Osimertinib is to selectively inhibit sensitive mutation and T790M mutation, effectively reduce the incidence of adverse reactions, and has high clinical safety.^28^

### Limitations of this study

It belongs to a single center study with small sample size and short follow-up time. In the follow-up study, multi centers, small large sample size and longer follow-up time should be considered.

## CONCLUSION

As the third generation EGFR-TKI new drug, Osimertinib has the same clinical effect as Gefitinib in the treatment of non-small cell lung cancer complicated with EGFR gene mutation, but PFS is significantly longer than Gefitinib group, and the adverse reaction rate is lower, which is worthy of clinical application.

### Authors’ contributions:

**XL & HZ:** Conceived and designed the study.

**ZZ & YZ:** Collected the data and performed the analysis.

**XL & HZ:** Were involved in the writing of the manuscript, are responsible for the integrity of the study.

All authors have read and approved the final manuscript.
